# Outcome of Er, Cr:YSGG laser and antioxidant pretreatments on bonding quality to caries-induced dentin

**DOI:** 10.1186/s12903-024-05390-4

**Published:** 2025-01-15

**Authors:** Lamiaa M. Moharam, Haidy N. Salem, Ahmed Abdou, Rasha H. Afifi

**Affiliations:** 1 Restorative and Dental Materials Department, Oral and Dental Research Institute, National Research Centre, Dokki, Giza, 12622 Egypt; 2 Operative Dentistry Department, Faculty of Oral and Dental Medicine, Nahda University, Beni Suef, Egypt; 3 School of Dentistry, Newgiza University, Giza, Egypt; 4 Restorative Dentistry Department, Faculty of Dentistry, Universiti Malaya, Kuala Lumpur, Malaysia; 5 Conservative Dentistry Department, Faculty of Oral and Dental Medicine, Future University in Egypt, New Cairo, Egypt

**Keywords:** Sodium hypochlorite, Er, Cr:YSGG laser, Antioxidant application, Sound dentin, Caries-induced dentin

## Abstract

**Background:**

This study aimed to assess the influence of different pretreatment protocols and antioxidants application on the shear bond strength (SBS) of universal adhesive to sound (SoD) and caries-induced dentin (CID).

**Methods:**

One hundred and twenty posterior teeth had their occlusal enamel removed, then the specimens were divided into two main groups according to dentin substrates; SoD and CID, three subgroups according to pretreatments protocols control (no pretreatment), NaOCl-treated, and Er, Cr:YSGG-treated and two divisions according to antioxidant application (with and without sodium ascorbate (SA) application). All-Bond Universal (ABU) universal adhesives was applied in self-etch (SE) mode then resin composite discs were built. The specimens were stored in distilled water for 24-hr at 37°C before SBS testing. Three-way ANOVA and Tukey HSD tests were used for data analysis (a = 0.05).

**Results:**

6% NaOCl resulted in a significant reduction in SBS in SoD without antioxidant application. 10% SA application showed significant increase in SBS for 6% NaOCl group only in SoD. Laser application recorded a significantly higher SBS compared to 6% NaOCl group without or with antioxidant application, while 10% SA application revealed a significant increase in SBS for control group only.

**Conclusions:**

Er, Cr:YSGG laser irradiation followed by antioxidant application has the potential to enhance the bonding quality of both tested dentin substrates. NaOCl application has significantly compromised the bonding to SoD and CID substrates.

## Background

In the current literature there has been a paradigm shift towards minimally invasive approach, that is associated with the development of efficient adhesive systems and effective bonding procedures. Such approach aims to conservatively manage the carious dentin lesion to preserve the sound dentin. Carious dentin composes two layers; the inner affected dentin surrounded by the outer infected dentin. Upon absence of bacteria; the remineralizable caries-affected dentin layer, should be preserved in conservative cavity preparation. Caries-affected dentin has different physical and chemical features than sound dentin (SoD), and shows lower bond strength values [1]. Bonding to caries-affected dentin exhibits some challenges during bonding procedures. Structural modifications in caries affected dentin, such as collagen denature and reduced mineral content may hinder dentin hybridization and jeopardize the mechanical characteristics of bonded restorations. Furthermore, obstructed dentinal tubules can inhibit resin diffusion, and prevent resin tags formation. However, the low-mineralized inter-tubular dentin in caries affected dentin permits more profound etching [2]. However, in-vitro studies often struggle to replicate the complex features and structure of naturally occurring caries-affected dentin [3]. Various approaches have been proposed to artificially induce caries-like dentin lesions (CID), but they may not fully replicate the long-term, natural caries process. Despite these differences, Joves et al. 2013 [3] concluded that natural CAD and artificial CID of permanent teeth were superficially analogous regarding the intertubular nanohardness.

Sodium hypochlorite (NaOCl) is a common endodontic irrigant in root canal treatments owing to its bactericidal impact. Moreover, it can deproteinize both mineralized and demineralized dentin substrates. Though, it can improve the bond strength through deproteinization effect and the removal of weakly attached smear layer. However, depleted bond strength was reported due to the strong oxidizing potential of the NaOCl [4], that could be owed to the release of NaOCl by-products that display an adverse influence on the polymerization of dental adhesives. However, such depleted bond strength values of NaOCl-treated dentin can be reinstated by application of antioxidant solution before the bonding procedures [5], as it is able to counteract the effect of NaOCl by-products and reversing the oxidizing effect of NaOCl on dentin surface [6].

Sodium ascorbate (SA) is an eminent antioxidant agent that can reduce free radicals. It was reported that SA can counteract the negative effect of NaOCl and peroxides on dentin bond strength through neutralization of their by-products. It was concluded that the depleted bond strength can return to normal by reduction of the oxidized dentin with a biocompatible and neutral antioxidant, such as SA, prior to the bonding procedures [7].

Erbium, chromium: yttrium-scandium-gallium-garnet (Er, Cr:YSGG) is one of the recent technological advances in dentistry. During the last few years, there has been a dramatic increase in laser applications for soft and hard dental tissues. Alternate treatments such as laser therapy using Nd:YAG and Er, Cr:YSGG demonstrated positive outcomes in bond strength enhancement. As both create a rough surface that is similar to the acid etching patterns. Thus, improve resin composite bonding to dentin [8].

Therefore, the objective of the current study is to assess different pretreatment approaches of SoD and caries-induced dentin (CID) using NaOCl and Er, Cr:YSGG laser prior to antioxidant agent application on SBS of a universal adhesive to the two dentin substrates. The null hypotheses tested were; 1- The different dentin substrates will have no effect on the bond strength pretreatment protocols. 2- The NaOCl and laser application will not affect the bond strength to different dentin substrates. 3- The application of antioxidant agent application will have no influence on the bond strength to different dentin substrates.

## Methods

### Ethical approval

This study was approved by the Research Ethics Committee of Oral and Dental Medicine, Future University (REC-FODM), New Cairo, Egypt; under the reference number: FUE.REC (13)/5-2-24. The authors declare that all conducted methods agreed with the guidelines and regulations of the ‘World Medical Association Declaration of Helsinki in 2013. The tested human teeth were extracted from anonymous participants for orthodontic purposes to be used for research objectives. The teeth were obtained from the outpatient clinic of the National Research Centre (NRC, Giza, Egypt). Informed consents were obtained from the participants for using their teeth samples in the study.

### Experimental design of the study

Sample size was calculated based on a pilot study of five samples to compare between different groups (Means= 9, 9.5 and 5.5, within subject SD=2). The effect size f=0.889 and a=0.05 resulting in minimum sample size of 8 in each and a 95% power. For statistical analysis reliability, the sample size was increased to ten teeth in each group.

**Fig. 1:**
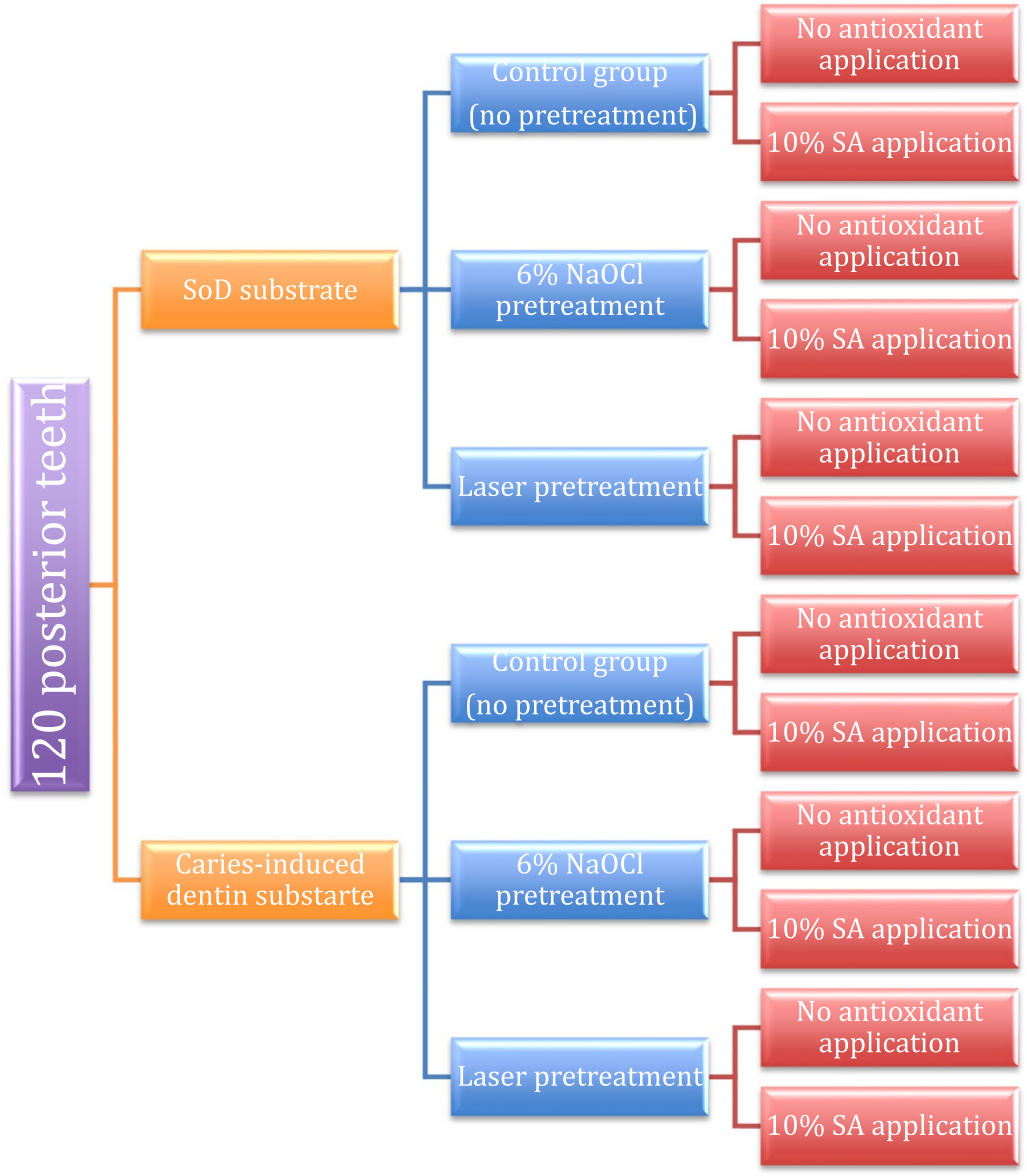
Experimental design, specimens’ grouping and frequency of the study

A total of one hundred and twenty premolar teeth were collected. Teeth divided into two main groups (*n*=60/each) based on dentin substrate type into: SoD and CID. Each main group was further divided into three subgroups regarding dentin pretreatment into: control without dentin pretreatment (*n*=20), NaOCl-treated dentin (*n*=20) and Er, Cr:YSGG laser-treated dentin (*n*=20). Then, each subgroup was finally alienated into two divisions according to antioxidant application into: no SA application (*n*=10) and 10% SA application (*n*=10). Figure 1 demonstrates specimens grouping, study design and the frequency.

### Selected materials

A universal adhesive; All-Bond Universal (ABU: BISCO Inc., Schaumburg, IL, USA), one cavity disinfectant; 6% NaOCl solution, and one antioxidant agent; 10% SA solution, and a nanofilled resin composite (Filtek™ Supreme Ultra: 3M Oral Care, St. Paul, MN, USA) were used in this study. Materials brand name, description, composition, and their manufacturers are listed in Table 1.

**Table 1:** Materials used in the study and their composition, description, and manufacturer

Material		Description	Composition	Manufacturer
All-Bond Universal (ABU)		Universal adhesive	10-MDP, ethanol, Bis-GMA, HEMA.	BISCO Inc., Schaumburg, IL, USA
Sodium Ascorbate (SA)		Powder	C6H7NaO6	Sigma-Aldrich, St. Louis, MO, USA
Filtek™ Supreme Ultra		Nanofilled resin composite restorative material	Bis-EMA, UDMA, TEGDMA, Bis-GMA, Zirconia particles (4-11nm nonagglomerated/aggregated and agglomerated), Silica clusters (20 nm non agglomerated/aggregated), Zirconia/silica aggregated particles (20nm silica particles and 4-11nm zirconia particles)	3M Oral Care, St. Paul, MN, USA

*Bis-EMA *Bisphenol A ethoxylate dimethacrylates, *HEMA *Hydroxyethyl methacrylate, *TEGDMA *Triethylene glycol dimethacrylate, *MDP *Methacryloyloxydecyl dihydrogen phosphate, *Bis-GMA *Bisphenol A diglycidyl methacrylate, *UDMA *Urethane dimethacrylate

### Teeth selection

One hundred and twenty human posterior teeth were collected for the current study. Any remaining soft tissues or debris were removed under tap-water using sharp hand scalers. A 25x magnifying lens was equipped to examine the selected teeth to eliminate any defective, fractured, or cracked teeth. Afterwards, the teeth were preserved in 0.1% thymol solution at 4°C up to a duration of three months maximum period post-extraction. The solution was changed once per week up until use [9].

### Specimens’ preparation

The roots of the selected teeth were removed 2-mm beyond the enamel-cementum junction using low-speed handpiece with mounted double-sided diamond cutting disc. Under wet condition, the occlusal enamel was ground flat with 240-grit silicon carbide (SiC) paper exposing the underlying dentin. Wet SiC paper of 600-grit was used to finish the exposed dentin surfaces for 60s in circular motion to develop uniform smear layer [10, 11]. Stereomicroscope (Olympus^®^ BX 60, Olympus Optical Co. LTD, Tokyo, Japan) was used to examine the specimens for enamel remains or additional flaws. Then the specimens were placed in blocks of auto cure acrylic resin [12]. After complete polymerization of the acrylic resin, the prepared specimens were stored in distilled water [13].

### Development of the caries-induced dentin (CID)

Artificially developed caries-induced dentinal lesions were produced through cariogenic challenge. Following the procedure proposed by Nicoloso et al. [14] as follows; two layers of an acid-resistant nail polish was applied to the specimens’ surfaces except for the exposed dentin surfaces. Specimens were then separately immersed in a demineralizing solution of adjusted pH= 4.5 (0.05 M acetic acid, 2.2 mM NaH2PO_4_, 2.2 mM CaCl_2_) for a duration of 8h, and then immersed in a remineralizing solution of adjusted pH= 7 (0.15 mM KCL, 0.9 mM NaH_2_PO_4_, 1.5 mM CaCl_2_) for 16h duration. The solutions were changed with fresh solutions and the specimens were thoroughly rinsed using deionized water then blotted dry, at the end of each cycle. This cycle was performed for 14d where the solutions were inspected intermittently using a pH meter. Half of the prepared dentin specimens (*n*=60) were exposed to a pH cycling protocol using prepared remineralizing and demineralizing solutions.

### NaOCl dentin pretreatment procedure

The respective prepared specimens were immersed in 6% NaOCl solution for 30s, followed by through rinsing with distilled water for 1-min to remove any residues of the solution [15].

### Er, Cr:YSGG laser dentin pretreatment procedure

According to Takada et. al [16], the respective specimens were treated with Er, Cr:YSGG laser system 2780nm (Biloase Technology Inc., San Clemente, CA, USA) using MZ8 tip of 800µm diameter, in a scanning motion on the occlusal surface for 30s with an output power of 2W, frequency of 20Hz and pulse duration of 140µs with 75% water coolant and 60% air coolant.

### Application of the antioxidant agent

Following the pretreatment procedures for different dentin substrates, the pretreated specimens were immersed in 10% SA solution for 10-min then rinsed thoroughly with distilled water for 1-min to remove any residues of the solution [15].

### Bonding procedures

The tested universal adhesive (ABU) was applied to SoD and CID specimens following SE bonding technique according to its manufacturers’ recommendations. ABU was actively applied in two coats with rubbing action to the pretreated dentin substrates using micro brushes for 10-15s per coat without light curing between the two coats [11]. Air syringe was used to evaporate the excess solvent by air-drying for ten seconds till there no visible movement of the adhesive was detected [9]. The tested universal adhesive was light-cured for 10s using LED light curing unit (=1000mW/cm^2^, Elipar S10, 3M ESPE, USA). The light curing unit was examined periodically using handheld radiometer (Demetron 100, Kerr Corporation, CA, USA).

### Resin composite application

Filtek Supreme Ultra nanofilled resin composite was used for composite discs build-up in one increment with the help of split Teflon molds of 2mm internal diameter and 2-mm height fixed over the dentin surface. Transparent celluloid strips were positioned on the top of the composite restorations. Each composite disc was light cured for 10s using the LED light curing unit according to the manufacturer’s instructions. Then the celluloid strips were removed and any flashes extending past the base of the composite discs were removed using a sharp blade. Then the specimens were reserved in distilled water in tight-seal plastic containers for 24-hr at 37°C until the SBS was evaluated [12, 13].

### Shear bond strength testing (SBS) and mode of failure assessment

The prepared specimens were attached to the lower jig of universal testing machine (Instron®, Model 3345, Instron Instruments, Buckinghamshire, UK). Chisel bladed metallic attachment mounted at the upper jig of the machine was placed as close as possible to the resin composite/dentin interface, and the test was run at 0.5mm/min cross head speed until failure with 5kN load cell. Maximum force was calculated in MPa. To calculate SBS, the peak load at failure was divided by the specimen’s surface area using the universal machine computer software (BlueHill® Universal, Instron Testing Software, Buckinghamshire, UK). The debonded specimens were examined using stereomicroscope at x35 magnification and modes of failure were classified as adhesive when the failure was located at resin composite/dentin interface, cohesive if the failure was identified within the resin composite or dentin substrates, and mixed when adhesive and cohesive fractures were acknowledged simultaneously.

### Statistical analysis

Shapiro-Wilk showed a normal distribution of the SBS, and three-way ANOVA test was used to demonstrate the effect of dentin substrate [SoD vs. CID], dentin pretreatment [Control (no pretreatment), NaOCl, and Laser application], and antioxidant application [No antioxidant application vs 10% SA application] on the shear bond strength. Tukey HSD was used for multiple comparisons. Statistical analysis was performed with IBM SPSS Statistics Version 20 for Windows (IBM Documentation products, Armonk, NY, USA).

## Results

Mean and standard deviation (SD) values [95% CI] for the SBS of different dentin substrates were demonstrated in Table 2. Three-way ANOVA test revealed that different dentin substrates, dentin pretreatment, and antioxidant application resulted in a significant effect on SBS at p?0.001. The interaction between the three variables resulted in an insignificant effect on SBS at *p*=0.156. For SoD substrate, 6% NaOCl resulted in a significant reduction in SBS compared to the control group and laser group without antioxidant application. On the other hand, 10% SA application resulted in a significant increase in SBS for 6% NaOCl group only. For CID substrate, Laser application resulted in a significantly higher SBS compared to 6% NaOCl group without or with antioxidant application. Meanwhile, 10% SA application revealed a significant increase in SBS for control group only.

**Table 2:** Mean and SD values [95% CI] for SBS of different dentin substrates

		Control	6% NaOCl	Er, Cr:YSGG Laser	*p*-value
**SoD**	No antioxidant	9.8±1.3 [8.4 to 11.1]^aA^	5.1±2.6 [2 to 8.3]^bB^	11±3.3 [7.5 to 1.4]^abA^	0.005
	10% SA	10.7±1.6 [9.1 to 12.3]^aAB^	9±1 [8 to 0.4]^aB^	12.7±2.9 [9.7 to 1.2]^aA^	0.019
**CID**	No antioxidant	4.2±2.3 [1.8 to 0.9]^bAB^	3.4±1.9 [1.5 to 0.8]^bB^	7.5±3.7 [3.7 to 1.5]^bA^	0.046
	10% SA	9.2±2.2 [6.9 to 0.9]^aA^	5.4±0.9 [4.4 to 0.4]^bB^	8.3±2.6 [5.5 to 1.1]^abA^	0.013
***p*-value**		<0.001	<0.001	0.034	

Different lowercase letters indicate significant different within column, while different uppercase letter indicate significance within rows (adjusted *p*-value with Tukey HSD)

**Fig. 2:**
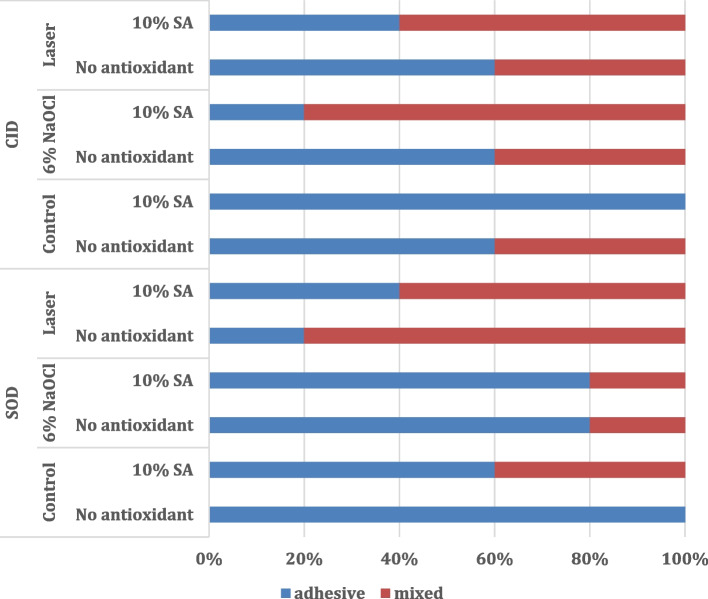
The modes of failure of the different tested groups of the study

**Fig. 3:**
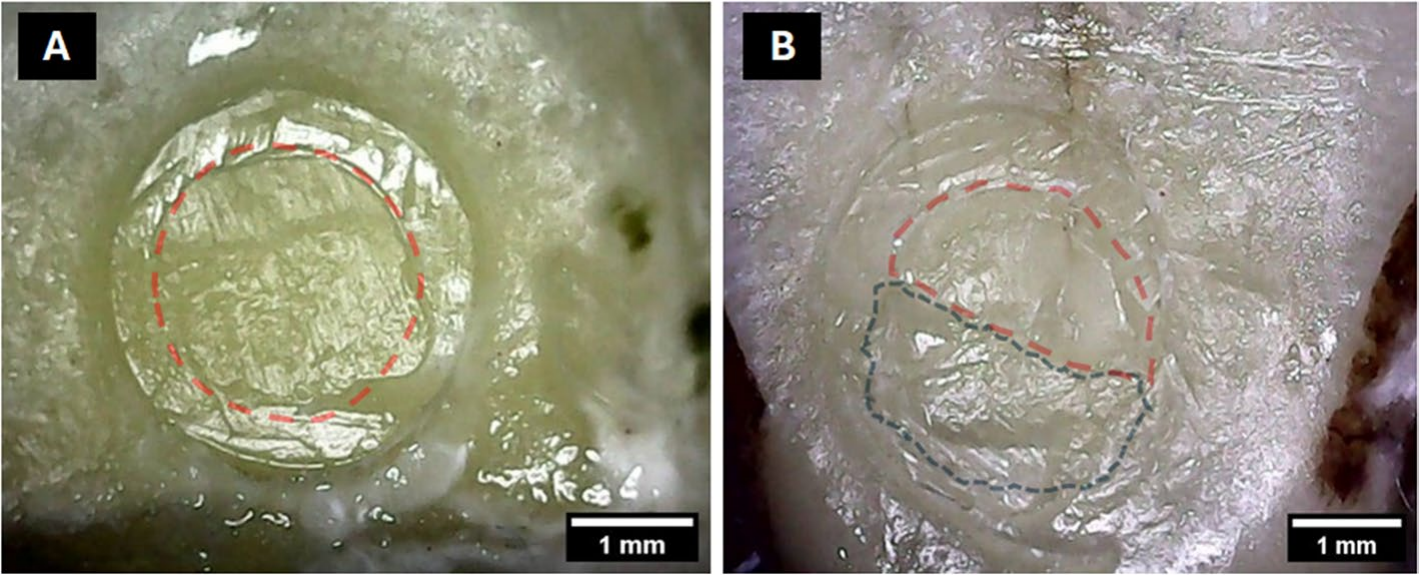
Representative stereomicroscope image for (**A**) adhesive failure [Red dashed line indicates area of adhesive failure] and (**B**) mixed failure [Blue dashed line indicates area of cohesive failure; Red dashed line indicates area of adhesive failure)

Failure mode results are presented in Fig. 2. For SoD substrate, the control group without antioxidant application showed 100% adhesive failure, while after 10% SA application, the results showed 60% adhesive failure and 40% mixed failure. 6% NaOCl groups, showed 80% adhesive failure with and without antioxidant application. For the Laser group, adhesive failure showed 20% without antioxidant application and 40% after 10% SA application. For CID substrate, the control group without antioxidant application showed 60% adhesive failure, while after 10% SA application, a 100% adhesive failure resulted. The 6% NaOCl group, showed 60% adhesive failure without antioxidant application and 20% with 10% SA application. For the Laser group, adhesive failure was 60% without antioxidant application and 40% after 10% SA application. Representative images of failure mode are presented in (Fig. 3).

## Discussion

The primary objective of minimally invasive dentistry is to thoroughly remove infected dentin while safeguarding healthy dental structures and eradicating cariogenic bacteria and decayed tissues prior to restoration, with notable similarities in mineral loss between caries-affected dentin and artificially induced dentin caries, yet distinguished by their formation processes, as natural caries exhibit two layers: a soft, bacteria-laden layer and a partially demineralized layer retaining collagen and viable odontoblasts, facilitating potential remineralization [17]. Lenzi et al. [18] proposed that chemically induced caries (CID) share similarities with natural acute caries lesions. To replicate artificial caries in dentin, researchers frequently use pH-cycling models, which are particularly useful for assessing bond strength across different tooth types [18]. Although chemical models can imitate tooth decay by alternating demineralization and remineralization phases, the exact duration of these cycles in the oral environment is not well established [19]. Nonetheless, pH-cycling procedures have been found to produce surface hardness comparable to natural decay in primary teeth, with lesions extending up to 40 µm deep [20].

Different dentin pretreatment regimes have been proposed such as applying cavity disinfectants such as NaOCl which can dissolve the collagen fibrils, promoting dentin deproteinization making the surface rich in apatite [21]. In addition, NaOCl dentin pretreatment will produce surface microporosity and irregularities, creating more permeable dentin surface that enhance adhesive monomer infiltration during the bonding procedures. Likewise, NaOCl dentin pretreatment can modify its ultrastructural morphology, which will lead to a significant increase in dentin wettability, dentinal tubules diffusion length, and number of resin tags and lateral branching [22]. However, cavity disinfectants may affect the hybrid layer, thus negatively influencing the restoration/dentin bond quality [18] by changing or loss of such layer leading to evident reduction in the bond strength that might results in increased rates of restorations failure. Consequently, many alternative treatment approaches have been advocated, like application of different remineralizing agents, antioxidants and laser treatment [8].

Dentin pretreatment with erbium laser enhances the morphological features of the dentin surface and improves the bonding quality of the dentin/restoration interface [23]. Its mechanism contrasts with that of NaOCl, relying on the water and hydroxyapatite in dental hard tissues to absorb laser energy, resulting in "micro explosions" that facilitate the extraction of water from target tissues via energy absorption [24]. The application of Erbium laser treatment has been found to enhance the surface roughness of dentin and expand its surface area via the patent dentinal tubules, consequently facilitating the diffusion of adhesive monomers into these tubules while preventing smear layer formation, thereby providing ideal dentin surfaces for effective bonding with resin composites and refining bonding techniques [24–26].

Universal adhesives are the newest generation of dental adhesives. They can be applied in either total -etch (TE) or SE modes to tooth substrate. They exhibit less clinical steps and they are more user-friendly. Bonding to caries effected dentin revealed decreased bond strength values of adhesive systems, but bonding of universal adhesives to different dentin substrates requires more research [2].

In this context, the current study investigated the possible effect of different dentin pretreatment modalities including laser and antioxidant application on bond strength to two dentin substrates. The results of the current study revealed that dentin substrates (SoD and CID), dentin pretreatment (6% NaOCl and Er, Cr:YSGG laser application), and antioxidant application (No SA and 10% SA application) had significantly impacted the SBS at p?0.001. Bonding to SoD revealed significantly higher SBS result values than CID in all tested groups (with/without dentin pretreatment and antioxidant application). Therefore, the first null hypothesis was rejected. This outcome might be owed to the composition of CID, since it is partially demineralized around and within the collagen fibrils with much lower crystalline structure related to normal SoD. That might lead to much softer and porous dentin surface than SoD. Moreover, CID has occluded dentinal tubules with acid-resistant minerals that might be impervious to resin infiltration. Consequently, the final SBS could be negatively altered [9, 27]. The findings of the current study agreed with Ekambaram et al. [28], they concluded that lower bond strength values could be developed by different resin adhesive systems to caries-affected dentin substrates than SoD substrate.

Treatment of dentin substrate with potent oxidizing agents such as NaOCl, has been suggested as an approach to reduce the sensitivity of hybridization technique. Furthermore, numerous dental procedures commonly depend on using NaOCl due to its non-specific deproteinization effect. NaOCl can remove the uncapsulated collagen fibrils, thus facilitate the penetration of the resin adhesives within the treated dentin surface producing more permeable surfaces. However, the impact of removal of collagen fibrils on bond quality to dentin demonstrated variable outcomes [28]. The finding of the current study showed that both dentin pretreatment approaches had a significant effect on SBS to both dentin substrates. Accordingly, the second null hypothesis was partially rejected as there was a significant difference between laser- and NaOCl-treated groups, though, there was an insignificant difference between laser-treated groups and the control groups for both dentin substrates. Regarding NaOCl application, the results revealed a statistical negative influence the SBS of both dentin substrates compared to the control group (no dentin pretreatment). This consequence might be related to the detrimental effect of NaOCl that is a potent oxidizing agent responsible for demineralized collagen fibrils elimination within the formed hybrid layer [29, 30].

The influence of NaOCl dentin pretreatment on the final bond strength to dentin is controversial. NaOCl was reported to improve dentin bond strength due to its deproteinizing effect providing a proper mineralized matrix that can be bonded directly to the adhesive monomer, as well as formation of an infrequent dentin bonding mechanism known as ‘reverse hybrid layer’ at which NaOCl dissolves the exposed collagen fibrils in mineralized matrix of the etched dentin and create surface microporosity to enable the penetration of the adhesive monomer to create this significant layer [31]. On the other hand, NaOCl was reported in other research to deplete dentin bond strength. These results were owed to its deproteinizing effect which created dentin surface that is less amenable for bonding [32]. Dentin pretreatment with NaOCl can remove the collagen fibrils thus preventing a continuous hybrid layer creation, and the remnants of NaOCl on the treated dentin surface was reported to prevent the infiltration of adhesive monomers [33]. Moreover, NaOCl oxidizing action can interfere with the polymerization adhesive monomers polymerization [34]. Another report from Montagner et al. [22] demonstrated that deproteinization pretreatment using NaOCl has shown comparable bonding performance to conventional adhesive procedures, where dentin regions play a significant role in bond strength values.

In this context, the finding of the current study agreed with de Almeida et al. [35] who demonstrated that NaOCl can directly hinder the free-radical additional polymerization reaction of the resin monomers, due to the remnants of super-oxide radicals it releases that inhibit the polymerization of the resin, producing a significant depletion of the final bond strength [26]. Moreover, NaOCl retention inside the demineralized dentin might have negative impact on the resin/dentin interface [36].

In contradiction to these finding, the effect of NaOCl was found in some literature to improve the bond strength or enhance the mechanical and physical properties of the resin/dentin interface [37, 38]. Such literature owed their results to the ability of NaOCl to dissolve the majority of organic content, thus, forming a mineral-rich layer that would be simply penetrated by the resin monomers. Though, it was concluded in another literature that its effect is adhesive-dependent [27, 35]. However, such contradiction could be owed to the difference in NaOCl concentration, time of application, solution temperature and type of the substrate.

Moreover, Kunawarote et al. [39] reported that dentin pretreatment using 6% NaOCl solution for 5- and 15-s showed an insignificant impact on µTBS of Clearfil SE Bond, whereas a 30-s time of application has a substantial adverse influence on µTBS to dentin. These findings agreed with the current study and previous research that concluded that smear layer-covered dentin pretreatment with NaOCl solution for 30-s duration or more had an adverse effect on dentin bonding quality [40]. They owed such lower µTBS values to NaOCl-pretreated dentin to its oxidizing effect that causes production of chloramine-derived free radicals [41], which might compete with the free radicals produced during adhesive monomer activation, causing premature termination of the chain reaction and probably inadequate polymerization [42]. Furthermore, dentin bond strength might be affected by residual NaOCl trapped within the porosity of mineralized dentin [43].

These results could be explained by the predominant adhesive failure (80%) that was recorded for 6% NaOCl-treated/SoD groups regardless of antioxidant application. While, 6% NaOCl-treated/CID groups demonstrated 20% and 60% adhesive failure with and without antioxidant application respectively. These findings agreed with Gönülol et al. [7] and Dikmen and Tarim [15] who revealed that adhesive failure demonstrated with NaOCl-treated groups.

Ascorbic acid and its Na-salts are distinguished antioxidants that can reduce various oxidative compounds. They counter the depleting influence of NaOCl on the bond strength to dentin through reinstating the changed redox reaction of the oxidized dentin substrate. Consequently, using SA before the bonding procedures can restore the depleted bond strength to NaOCl-treated dentin [15, 44]. The results of the present study revealed an improved SBS values after different dentin pretreatments of both dentin substrates following 10% SA application. Thus, the third null hypothesis was partially rejected as a comparable bond strength value was recorded for the two tested dentin pretreatments for both dentin substrates with and without antioxidant application. The application of 10% SA showed a significant increase for NaOCl-treated groups in SoD. This consequence could be attributed to the effect of antioxidant application that might have counteract the potent oxidizing effect of NaOCl. It was concluded that, antioxidant agents like SA can effectively nullify the reactive oxygen produced during the oxidation process of NaOCl with dentin [45–47]. These findings were in accordance with Prasansuttiporn et al. [48] who concluded that further application of different antioxidant agents following deprotenization of the smear layer by NaOCl could enhance the final bond strength values of a SE adhesive to caries-affected dentin. Moreover, Delgado et al. [30] stated that without using an antioxidant agent for NaOCl-treated dentin, the outcomes cannot be validated, due to poor bond strength outcomes that could be accredited to NaOCl oxidizing effect that adversely influences resin polymerization instead of the treatment approach itself, and the depleted bond strength could be retrieved through application of antioxidant agents to NaOCl-treated dentin. Likewise, Dikmen and Tarim [15] demonstrated that using 10% SA for NaOCl-treated dentin surface had significantly enhanced dentin bond strength. Gönülol et al. [7] demonstrated the capability of SA to restore the depleted bond strength of oxidized dentin by allowing the additional polymerization of the resin adhesive to progress without early termination, thus, reversing the jeopardized bonding in NaOCl-treated dentin.

Due to the recent advances in modern dentistry, different types of laser devices have been introduced to the market. Amid the diverse laser devices, Er,Cr:YSGG that is operated at 2780 nm wavelength. It is properly absorbed by different biological tissues such as enamel and dentin. The primary objective of the diverse types of laser devices is alteration of light energy of laser devices into heat leading to a substantial increase in laser energy absorption by the substrate. The degree of absorption of laser energy is affected by several surface characteristics that may include the extent of the irradiated surface pigmentation and its content of water [48]. The results of the current study displayed that the Er, Cr:YSGG laser-treated groups exhibited a significantly higher SBS values compared to NaOCl-treated groups, however, their results were comparable to the control groups (no dentin pretreatment) regardless the dentin substates and antioxidant application. This outcome could be generally related to the effect of Er, Cr:YSGG laser irradiation on the different tested dentin substrates, through the micro-explosions produced by evaporation of water and other moist organic components of dentin resulting in removal of the smear layer and irregularities development on the dentin surface and further opening of dentinal tubules. Thus, making the dentin surface more permeable and ideal for bonding [49]. Regarding this result, it was concluded that the removal of the laser-modified layer by etching has restored the depleted bond strength back to normal [50–52]. This outcome agreed with Celik et al. [53] and Ferreira et al. [54] who concluded that SE adhesives can enhance the bond strength of laser-irradiated dentin than etch-and-rinse adhesives. Moreover, this finding was in agreement with Alkhudhairy and Neiva [8] who concluded that using low-power Cr:YSGG laser improved the bond strength to CID substrate, by enabling the laser energy to interact with the water molecules of the dentin surface leading to their evaporation, which might lead to collagen fibrils shrinkage and dehydration [54]. This could result in decreasing the surface area of the dentin, thus permitting enhanced monomer infiltration and better dentin adhesion. Furthermore, such dehydration could alter the surface features of the dentin by changing it from hydrophilic to hydrophobic substrate with more enhanced bonding [8]. Generally, laser treatment showed no adverse effects on adhesion performance. The variance in outcomes among different the studies in laser-treated tooth surfaces, can be owed to numerous factors such as; the type of applied laser, the parameters of the used laser device including; distance of application, frequency, energy, and application mode as well as the applied adhesive system type [55]. On the other hand, these findings were contradicted by those attained by Al Habdan et al. [26] who reported a significant decrease in dentin bond strength after laser irradiation. This might be to using Er, Cr:YSGG laser with 4.5-W power output, that can be considered as a high value for laser pretreatment causing serious surface alterations, thus, preventing resin penetration and causing a substantial reduction in the final bond strength [56–58]. Vermelho et al. [57] reported that the application of laser has modified the bonding mechanism of the adhesives to dentin substrate decreasing the bond strength for SE adhesives, through integration of small-size and few particles of dentin developed during laser ablation into SE adhesive layer. Moreover, the tested laser settings had no impact on dentin SBS, regardless aging time and type of adhesive. In addition, Comba et al. [59] reported decreased bond strength values of Er:YAG laser-treated dentin regardless adhesive type. Such contradiction could be related to the use of Er:YAG laser instead of low-power Er, Cr:YSGG laser (2W) as in the current study. In this context, a few studies reported that erbium laser application has reduced the overall dentin bond strength to resin composite materials [60, 61]. Shirani et al. [61] concluded that erbium laser irradiation had decreased the overall SBS. They owed such finding to the irradiation distance which significantly affected the SBS values as decreasing the distance increased the adverse effects of laser irradiation. They demonstrated that irradiation distance presented an imperative parameter that it is directly associated with the laser ablation-ability, morphological features of the lased surface and the subsequent achievement of the bonding process [61]. While, Ceballos et al. [62] suggested that the collagen fibrils have been fused together following laser ablation of dentin, that might result in absence of interfibrillar space. Thus, adhesive monomer infiltration within the subsurface inter-tubular dentin would be hindered, lowering the final bond strengths regardless dentin substrate type and adhesive type. The inconsistent outcomes regarding the quality of bond strength to irradiated caries affected dentin substrates [59] could be owed to the impact of the wide variation in the parameters of laser irradiation, such as frequency, duration, output power and distance. Additionally, such contradicting findings could be associated with using different adhesive systems and lack of longitudinal study designs [63].

Consequently, the results of the study revealed that the application of SA after Er, Cr:YSGG laser irradiation for both dentin substrates has the potential to improve the bond strength. This could be explained by the combined positive effect of SA application and Er, Cr:YSGG laser irradiation on bond strength to dentin. As the Er, Cr: YSGG laser has the ability to change the characteristics of dentin surface through removal of smear layer with patent dentinal tubules, while SA can eliminate the oxygen at the dentin surface, so that oxygen absence at the bonding area might enhance the adhesive polymerization and thus, improving the final bond strength [51]. This was in accordance with Rezaei et al. [52] who concluded that antioxidant agents’ application can even improve the bond without bleaching.

Although, Er, Cr:YSGG laser and SA have different mode of action and characteristic potent effect on dentin, it seems a promising adhesive strategy to use laser treatment followed by antioxidant application for better dentin bonding. As the combined application of laser irradiation and antioxidant application on bond strength to different dentin substrates is not properly discussed in current literature, therefore, more research is required to validate these results.

Consequently, such findings could be related to the fracture mode analysis results that showed adhesive mode of failure with 40% for antioxidant application and 20% without antioxidant application for SoD groups, while adhesive failure was 60% without antioxidant application and 40% with antioxidant application for CID groups. These findings could be related to the thermo-mechanical ablation effect of Er, Cr:YSGG laser irradiation on both dentin substates, indicating that the SBS test was well-conducted, and no undesirable stresses were generated at the resin-dentin interface. These findings were in accordance with Ribeiro et al. [50] who demonstrated the absence of cohesive failure and predominance of adhesive failure followed by mixed type of failure. Moreover, Alrahlah [63] showed that adhesive failure comprised the majority of resultant failure types within the tested groups, denoting that the adhesive failure is considered favorable as it avoids any impractical harm or loss of the tested dental substrate. However, it was concluded that the debonded specimens after SBS testing showed that CID irradiated with Er, Cr:YSGG laser showed cohesive failures that are commonly related to high bond strength values that might be due to several external factors such as and microporosities within the adhesive layer, anatomy of dentinal tubules, level of tubular occlusion, degree of dentin remineralization and the dentin-binding ability of the adhesive [8].

The current study has some limitations, including the use of the shear bond strength (SBS) method instead of the micro-tensile bond strength (µTBS) method. While µTBS offers better control of regional differences and economic tooth use, it is more technique-sensitive and can be challenging to perform, especially with small specimens. Additionally, µTBS requires specimen trimming, which can lead to tooth cracking and premature failure if not done carefully [64, 65]. The SBS method was chosen for its simplicity and speed, making it popular in research settings. Although SBS may not always detect cohesive failure, it remains the most common method for evaluating new adhesive systems [66, 67]. In this study, the SBS test was conducted using an Instron universal testing machine with a chisel-bladed metallic attachment, providing a more feasible and affordable approach in the research lab.

There are other limitations of the study include the use of only universal adhesive system in SE mode and application of laser irradiation at a short duration. Also, employing other dentin substrates such as eroded and sclerosed dentin at different depths would be of more value. Hybrid layer assessment in aged specimens under the same conditions of the current study presents another limitation of this in vitro study. In this context, one can recommend to assess the effect of Er, Cr:YSGG laser irradiation at different parameters and NaOCl among other dentin pretreatments with different antioxidants application, on different cariogenic bacterial strains experimentally grown on dentin surface. Additionally, it would be worthy to assess diverse types of adhesive systems including SE and TE on different dentin substrates, and to evaluate their effect on the interfacial surface morphology and the hybrid layer using scanning electron microscope (SEM) to augment and validate the consequences and to overcome the confines of the present study. Likewise, investigating the durability and longevity of CID and SoD aged specimens bonded to different restorative systems would be of great value for further research.

## Conclusions

Under the limitations of the current study, it can be concluded that; pretreatment of different dentin substrates using Er, Cr: YSGG laser irradiation followed by antioxidant application has the potential to enhance the bonding quality of both tested dentin substrates. Nevertheless, using NaOCl for dentin pretreatment has significantly compromised the bonding to SoD and CID substrates regardless SA application. Moreover, restored SBS is a far-reach consequence of antioxidants application. SA application can improve the bond strength to different dentin substrates following different pretreatment protocols.

## Data Availability

The datasets generated during and/or analyzed during the current study are not publicly available due to institutional policy but are available from the corresponding author on reasonable request.

## Abbreviations

SBS: Shear bond strength
SoD: Sound dentin
CID: Caries-induced dentin
Er,Cr:YSGG: Erbium, chromium: yttrium-scandium-gallium-garnet
Nd:YAG: Neodymium-doped yttrium aluminum garnet
SE: Self-etch
NaOCl: Sodium hypochlorite
SA: Sodium ascorbate
ABU: All-Bond Universal
CAD: Caries-affected dentin
REC-FODM: Research Ethics Committee of Oral and Dental Medicine, Future University
FUE: Future University of Egypt
HEMA: 2-Hydroxyethyl methacrylate
Bis-GMA: Bisphenol A glycerolate dimethacrylate
MDP: Methacryloyloxydecyl dihydrogen phosphate
UDMA: Urethane dimethacrylate
TEGDMA: Triethylene glycol dimethacrylate
Bis-EMA: Bisphenol A ethoxylate dimethacrylates
SiO_2_: Silicon dioxide
SiC: Silicon carbide
MPa: Megapascals
N: Newtons
SD: Standard deviation
ANOVA: Analysis of variance
LED: Light-emitting diode
TE: Total-Etch
NRC: National Research Centre
µTBS: Micro-tensile strength
